# Effect of Different Types of Electrospun Polyamide 6 Nanofibres on the Mechanical Properties of Carbon Fibre/Epoxy Composites

**DOI:** 10.3390/polym10111190

**Published:** 2018-10-25

**Authors:** Cristina Monteserín, Miren Blanco, Nieves Murillo, Ana Pérez-Márquez, Jon Maudes, Jorge Gayoso, Jose Manuel Laza, Estíbaliz Aranzabe, Jose Luis Vilas

**Affiliations:** 1 Unidad de Química de superficies y Nanotecnología, Fundación Tekniker, Iñaki Goenaga 5, 20600 Eibar, Spain; miren.blanco@tekniker.es (M.B.); estibaliz.aranzabe@tekniker.es (E.A.); 2 Smart System Area, TECNALIA, P Mikeletegi 7, E-20009 Donostia-San Sebastian, Spain; nieves.murillo@tecnalia.com (N.M.); ana.perez@tecnalia.com (A.P.-M.); jon.maudes@tecnalia.com (J.M.); jorge.gayoso@tecnalia.com (J.G.); 3 Departamento de Química Física, Facultad de Ciencia y Tecnología, Universidad del País Vasco/EHU, Apdo. 644, E-48080 Bilbao, Spain; josemanuel.laza@ehu.eus (J.M.L.); joseluis.vilas@ehu.eus (J.L.V.)

**Keywords:** carbon fibres, polymer matrix composites (PMCs), delamination, fracture toughness, electrospun nanofibres

## Abstract

Delamination and brittle matrix fracture have long since been the biggest problems in fibre-reinforced composites. Recently, the incorporation of electrospun nanofibre veils has been shown to be an effective method for improving the mechanical properties of these composites, without causing process problems and negatively affecting other mechanical properties. Thus, these nanofibres have the potential to be used as thickness-reinforcing materials in composites. This paper investigates the effect of incorporating standalone electrospun nanofibre veils made of two different types of polyamide 6 (PA6) on the mechanical properties of carbon fibre/epoxy composites. The influence of positioning the electrospun veils at different interlaminar positions of the laminate has also been investigated.

## 1. Introduction

Fibre-reinforced epoxy resin composites are widely used in industry, owing to their high strength and stiffness at low weight, and their good corrosion resistance and fatigue properties. However, epoxy matrices are brittle materials, which could lead to the unexpected failure of the composite [1].

Great research efforts have been made to prevent composite failure by the modification of the epoxy matrix, either by chemically modifying the resin and hardener components, e.g., by using dendritic hyperbranched polymers [2], or by adding a second dispersed component to the epoxy resin [3,4,5]. The second phase can be a particulated phase such as preformed particles, inorganic or polymeric particles [3], or can be a rubber or a thermoplastic material that is initially soluble in the epoxy resin, which could phase-separate during the curing of epoxy matrices to form the particulated phase [4,5]. Although this second phase inside of the epoxy matrix often increases the fracture toughness of the laminates, this increase could be accompanied with an increase in the laminate thickness and a decrease in the glass transition temperature or in the other mechanical properties of the composite, such as stiffness and strength [6]. Nanoparticles can also be added to the epoxy matrix to improve its mechanical properties [7,8,9,10,11,12,13]. Due to their theoretical high stiffness and strength, nanoparticles might improve the matrix characteristics [8,9]. However, the overall improvement in mechanical properties such as the stiffness and fracture toughness of the epoxy matrix is mostly very moderate [3,10,11,12,13]. The main disadvantage with nanomaterials, besides the safety issues due to their small dimensions [13,14,15,16], is the difficulty in obtaining a homogeneous dispersion of the nanoparticles in the resin matrix and the viscosity increase of the resin when nanoparticles are added, limiting its use in infusion applications [2].

Recently, the use of electrospun thermoplastic nanofibres veils has emerged as a promising technique to toughen laminated composites without deteriorating the in-plane mechanical properties and without significantly increasing the laminate thickness and weight [17,18,19,20,21,22]. Electrospun nanofibres can be easily deposited on carbon [18,22,23,24,25,26,27] and glass fibre fabrics [16,28] or can be produced as standalone structures [16,17,18,19,28,29,30,31,32], which are called veils. Both ways of nanofibre incorporation have no negative effect on the impregnation of the epoxy resin in the fabric reinforcement. Therefore, it can be easily adapted to traditional composite structures’ manufacturing techniques as an independent product or as an additional layer of fabric reinforcement.

Thermoplastic nanofibrous structures offer a solution for the dispersion and viscosity issues that are detected in the case of particulate addition in the resin, since they can be readily embedded in the resin and incorporated as a homogeneous nanosized phase in the laminated composite. The high porosity of the nanofibrous non-woven network enables easy impregnation with epoxy or even thermoplastic matrix materials [33]. Moreover, due to their macroscale length, the diameter is nanometric, but the length is continuous for the macroscopic dimensions of the veils because of its non-woven network structure; no health hazards concerns are involved in the production and use of these nanofibres. Recent literature has indicated that nanofibres may contribute substantially to the ductility and fracture toughness of the composites [22,28]. This is related to the hypothesis that a secondary fibrous structure with a pronounced lower fibre diameter besides a primary fibre structure may increase several of the mechanical properties of composite materials.

Electrospun nanofibres veils that are used as interleaving reinforcement offer the possibility of customised veils structure and nature through their area density or a porous/fibres ratio customisation, as well as the use of a broad number of thermoplastic polymers or blending. These technical kindnesses, together with the low-cost nature of the manufacturing process and their friendly implementation into existing composite manufacturing processes, make the electrospinning process a good candidate for the manufacture of interleaving reinforcement veils.

The present work studies the effect of the incorporation of electrospun polyamide 6 (PA6) nanofibre veils coming from two different types of pellets in the final mechanical properties of carbon fibre epoxy composites with the objective of studying the influence of the polyamide 6 (PA6) mechanical properties and nature on the composite material. The nanofibrous veils are incorporated into different positions of the laminate in the composite as standalone structures, and then, carbon fibre epoxy composites are manufactured by a vacuum infusion process. To investigate the mechanical properties of the nanofibre veil-reinforced composites, flexural tests, dynamical mechanical analysis, and mode I and mode II tests were carried out on the reference and nanofiber-reinforced composite specimens. The crack propagation in the loaded sample was studied through scanning electron microscopy. This research will contribute to increasing knowledge about fibre composites modified with electrospun veils and wider acceptance of these materials in the composite industry.

## 2. Materials and Methods 

### 2.1. Materials

As a matrix, a difunctional diglycidyl ether of bisphenol A (DGEBA) resin was employed. The resin, which is known as Epikote 828, has been supplied by Momentive (Waterford, NY, USA), and presents an equivalent weight of 182–190 g·equiv^−1^ and a hydroxyl/epoxy ratio of 0.03. As a curing agent, 4,4′-diaminodiphenylmethane (DDM) from Alfa Aesar (Lancashire, UK), was employed. DDM is a solid tetrafunctional aromatic amine with a molecular weight of 198 g/mol and an amine equivalent weight of 49.5 g·mol^−1^. For the electrospun nanofibre preparation, two PA6 pellets with different mechanical properties were employed, Badamid^®^ B70 from BADA AG (Bühl, Germany), and Ultramid^®^ B24 N 03 from BASF SE (Ludwigshafen, Germany). The PA6 pellets were dissolved in a 2:1 mixture of formic acid: acetic acid, both from Panreac Química SLU (Barcelona, Spain) for the electrospun solution preparation. The carbon fibre fabric employed for the composite manufacture was a plain wave bidirectional woven mat with 200 g/m^2^, which was formed by HT3k fibres from SP Systems (Newport, UK).

### 2.2. Sample Preparation

For the development of the nanofibre veils, 12 wt % of both types of PA6 pellets were dissolved in the 2:1 mixture of acetic acid:formic acid by stirring for two hours at 80 °C. To obtain large uniform nanofibrous structures, the nanofibres were produced using a multijet electrospinning setup Nanospider™, from Elmarco, using a high-volume spinning tub. The solution is poured into the feed unit, and a cylindrical electrode formed by six wires is placed in the middle of the solution tank. The feed unit is moving back and forth, and the solution is feeding on the wire. The upward part has a second wire electrode, which has the opposite charge to the downward wire electrodes. The electrical field between the electrodes overcomes the surface tension of the polymer solution, the thousands of jets that are formed then become fibres when the solvent is evaporated, as the fibre flies between the downward wires and the forthward collecting electrode. An antistatic dielectric supporting material is placed between the flying fibres and the forthward electrode as substrate material for the nanofibres’ collection. The dielectric supporting material is moving at a desired speed using take-up cylinders. All of the non-woven nanofibrous were spun in a conditioned room at 23 ± 2 °C and 50 ± 5% RH. The distance between the collector and the cylinder was 170 mm, and the spinning rate was set at 6.7 rpm. The voltage was adapted until a stable process was achieved; this was at 75 kV. The standalone structures were electrospun onto antistatic polypropylene. The electrospinning time was 60 min.

In order to gain a better understanding of the effect of the inclusion of nanofibrous veils in the final mechanical properties of laminated composite materials, conceptual composites formed by two carbon fibre plies positioned at 0° and interleaved with standalone nanofibrous veils were prepared. Two configurations for positioning the PA6 veils have been considered: in configuration 1, a PA6 veil was integrated in the interlaminar region, and in configuration 2, PA6 veils were integrated not only into the interlaminar region, but also in the external layers of the composite. The composite laminate plates were fabricated by infusion moulding. DGEBA epoxy resin was placed in an oven at 80 °C overnight to remove any water present. Then, epoxy–amine formulations were prepared by stirring the stoichiometric mixture among the DGEBA resin and the diamine vigorously for 10 min at 80 °C [11,12]. The liquid resin was forced into the laminate by the pressure difference between the atmospheric pressure acting on the resin and the level of vacuum in the composite laminate. The liquid resin was infused through the whole laminate, not observing differences in impregnation using PA6 Ultramid or Badamid veils. In the configuration with three veils, the infusion process was slower. The system was cured at 90 °C for four hours, which is a conventional curing cycle in the composite industry, ensuring that the veils do not melt during curing. The incorporation of polyamide nanofibre veils did not affect the curing kinetic of the composite. The resulting composite laminate was a homogenous structure of 30 × 30 cm, with all of the components bound together within the resin matrix. The samples were cut to the dimensions required for the different experimental analysis with a multi-use DREMEL 4000JF. All of the specimens that were prepared with two carbon fibre layers had thicknesses between 0.6–0.7 mm. For the fracture test (mode I, mode II), composites with 14 layers of carbon fibre and an interlaminar veil in the central axis were also developed using the vacuum infusion technique; these had thicknesses between 3.1–3.6 mm.

### 2.3. Characterisation Techniques

Fourier transform infrared spectroscopy (FTIR) spectra were obtained using a FT/IR 4700 (JASCO, Easton, MD, USA) equipment accomplished with an attenuated total reflection accessory PIKE GladiATR (Pike Technologies, Madison, WI, USA). Twenty scans were taken for each sample with a resolution of 4 cm^−1^ in the range of 400 cm^−1^ to 4000 cm^−1^. Differential scanning calorimetry (DSC) measurements were performed in a DSC1 module from Mettler-Toledo (Gießen, Germany) equipped with an intracooler and previously calibrated with high-purity indium and zinc standards. Differential scanning calorimetry has been used to characterise both the veils and the composite specimens. For the characterisation of the veils, experiments were performed at two successive temperature heating scans from 0 °C to 300 °C at 10 °C·min^−1^. For the composites, the test range was 25 °C to 320 °C at a constant rate of 10 °C/min. All of the measurements were conducted in a dry atmosphere under a constant nitrogen flow of 50 mL/min, working with samples between 7 and 10 mg. Thermogravimetric analysis (TGA) was carried out via a thermogravimetric analyzer SDT Q600 (TA instruments, New Castle, DE, USA). The analysis was carried out from ambient temperature to 800 °C, at a heating rate of 10 °C/min, under a nitrogen atmosphere (100 mL/min).

Scanning electron microscopy (SEM) and a Carl Zeiss SMT Ultra Gemini-II system (Carl Zeiss Microscopy, LLC, Thornwood, NY, USA) were used to investigate the cross-sections of the composite plates. Dynamic mechanical thermal analysis (DMTA) measurements were performed on the samples of composites with the two different PA6. A Polymer Laboratories Mark II DMTA instrument (Agilent Technologies, Santa Clara, CA, USA) was used to analyse the composites. Rectangular samples (10 mm × 35 mm) were directly cut from the composite sheets and measured using the dual cantilever test from −50 °C to 200 °C, at a heating rate of 2 °C/min, 64-µm strain, and at several deformation frequencies (1 Hz, 3 Hz, and 10 Hz). Samples of the reference system were also measured in the same conditions.

The flexural properties were obtained from three-point bending tests performed on rectangular specimens (60 mm × 20 mm) using an electromechanical Instron 3369 machine (Instron, Norwood, MA, USA) with a load cell of 1 kN. This size has been selected to avoid the slippering of the specimens during the test. The crosshead speed was 5 mm/min. The Mode-I interlaminar fracture toughness of the composites was determined by double cantilever beam (DCB), following the AITM 1.0005 standard [34]. In each test, the specific thickness value was considered. The dimensions of the DCB test specimens were 250 mm in length and 25 mm in width. Aluminum blocks were bonded to the outer surfaces of the DCB specimens to transfer the opening forces. An initial crack of 25 mm was generated. Then, the specimen was loaded until the crack propagated about 100 mm from the tip of the crack, following the guidelines of the standard. Three specimens have been tested using a test speed of 10 mm/min. Load, opening displacement, and crack length were recorded for the energy release rate (G_IC_) calculation during the tests. The mode-II interlaminar fracture toughness of the composites was determined by end-notched flexure experiments, which consist of a three-point bending experiment on specimens with an initial delamination. In mode II, the test specimen is loaded by shear stresses, which are introduced by a bending moment. The fracture of the interface occurring during this test is an in-plane shear fracture. For the determination of a quantitative value of the fracture energy of the interface, the load and displacement during the test are recorded. Using the maximum load and displacement from the plot, the fracture toughness energy of the interface (G_IIc_) is calculated. The specimens employed in the analyses are the same three specimens that are tested in mode I.

## 3. Results and Discussion

### 3.1. Characterisation of the Electrospun Polyamide 6 Nanofibre Veils

**Figure 1:**
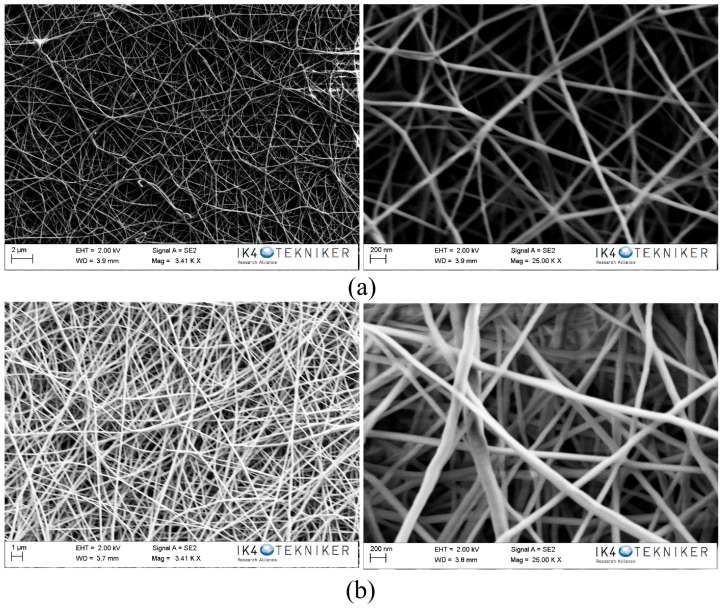
SEM images of polyamide 6 (PA6) electrospun nanofibres: (**a**) Ultramid and (**b**) Badamid.

Figure 1 shows the SEM images of the PA6 Ultramid and PA6 Badamid nanofibres. A continuous and uniform nanofibre network without bead formation was obtained using the selected electrospinning working parameters. Depending on the type of polyamide, the average nanofibre diameter was between 60–100 nm for PA6 Ultramid (Figure 1a) and 60–130 nm for PA6 Badamid (Figure 1b).

The areal weight density of the electrospun nanofibres has been determined by weighing a 10 cm × 10 cm area in a balance with an accuracy of 0.0001 g. Table 1 summarises the values obtained for the areal weight density and fibre diameter of the veils. The weight difference between the different polyamides is related to the diameter of the nanofibres. In this case, the diameters of the fibres obtained for both Ultramid and Badamid are similar, so the weights obtained are quite similar, and are slightly larger in the case of the PA6 Badamid.

Infrared spectroscopy in attenuate total reflectance (FTIR-ATR) was used to characterise the PA6 electrospun nanofibers. The characteristic PA6 absorption bands were all detected, and the spectra obtained for both samples were quite similar [35].

Thermogravimetric analysis (TGA) of the electrospun nanofibres was performed. Both Ultramid and Badamid presented a significant loss of mass at temperatures close to 400 °C, corresponding to the degradation of the carbonaceous structure. After the analysis, a residue of 5 wt % corresponded to the presence of carbon char or carbonaceous material that could not be dissociated into smaller volatile fragments and was maintained at high temperatures. It can also be confirmed that the nanofibres did not present an excess of solvent as a result of the electrospinning process.

**Figure 2:**
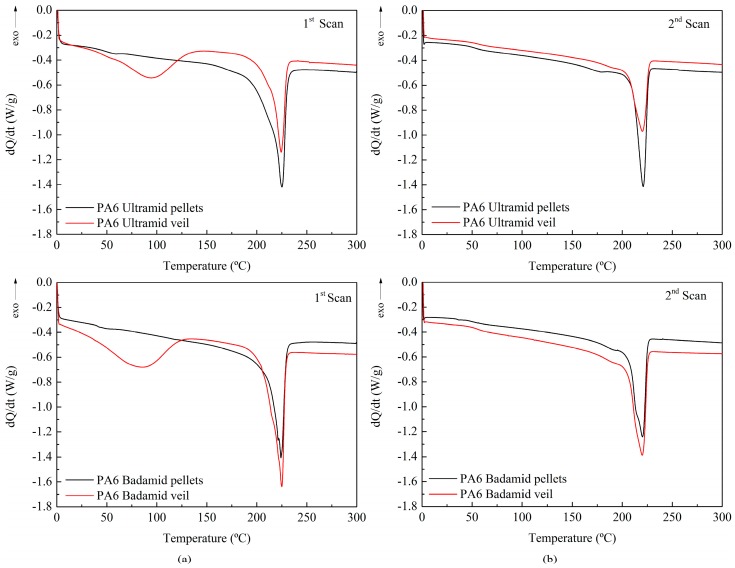
Differential scanning calorimetry (DSC) dynamic thermogram of the PA6 Ultramid and PA6 Badamid pellets and electrospun nanofibres (**a**) first scan and (**b**) second scan.

DSC was used to determine the thermal properties of both the pellets and the electrospun nanofibres of PA6 Ultramid and Badamid. Figure 2 shows the first and second heating DSC scans for the PA6 Ultramid and PA6 Badamid pellets compared with the corresponding electrospun nanofibres. Table 2 shows the melting and the crystallinity for both PA6 from the first and the second DSC scans. The crystallinity was calculated using Equation (1):

(1) $$\%\;\mathrm{Cristallinity}=\frac{\Delta H_{m}}{\Delta H_{m}^{f}}\times 100$$

where *ΔH*_m_ is the melting enthalpy of each sample, and *ΔH*_m_*^f^* is the melting enthalpy of theoretical 100% crystalline PA6, which is 230 J·g^−1^ [36].

In the first DSC scan, both PA6 Ultramid and Badamid pellets and electrospun nanofibres show a single melting peak almost between 224–225 °C with a slight widening at lower temperatures. This indicates the presence of a higher proportion of PA6 in α-form and a slight content of γ-form, whose melting temperatures appear at approximately 220 °C and 210 °C, respectively.

The enthalpy of fusion is greater in the pellets, which indicates that a lower degree of crystallinity has been achieved in the veils than in the initial product. In the PA6 Ultramid veils, the crystallinity decreased significantly from 46.5% to 26.6%, while for the veils obtained from PA6 Badamid, the crystallinity slightly decreased from 41.5% to 38.2%. In the second scans, it is observed that the melting temperatures of the veils and their respective pellets were also very similar among them, although these temperatures were lower than those obtained in the first scan. This reduction may be due to morphological effects, since crystals of lower quality may have formed in the crystallisation process that occurred after the first heating. In this second heating, the enthalpies obtained for the pellets and for the veils were similar, obtaining degrees of crystallinity in the order of 25% for PA6 Ultramid and 34% for PA6 Badamid. These values confirmed the highest degree of crystallinity of the veils obtained from PA6 Badamid. It is demonstrated that the high crystallinity of the PA6 Ultramid pellet that was observed in the first scan was due to the thermal history of the pellets and, therefore, this characteristic could not be transferred to the veils that were obtained from it. In addition, the curves corresponding to the first scan of the veils showed a thermal process in the temperature range between 30–125 °C, which did not occur in the pellets. Due to the masking effect of this peak, the *T*g of the PA6 is not observed, which normally appears in the range of 45–65 °C for these samples, and which is clearly seen in the DSC curves for the pellets in the first and the second scan of both materials. There is much controversy surrounding the assignment of this thermal process at low temperature. Many authors have associated it with the evaporation of residual solvent or water trapped inside non-woven nanofibres [37,38]. Other authors have related it to the electrospinning process itself [39,40]. Since no significant mass loss was observed during the TGA, it was assumed that there was no presence of volatile products, such as solvents, from the electrospinning process. Therefore, it would seem logical to attribute this enthalpy corresponding to an endothermic process to the melting of small crystals that were formed during the electrospinning process [41,42].

To understand the influence of the veil on the performance of the composites, they were characterised by dynamic–mechanical tests, bending and fracture tests, and SEM.

### 3.2. Characterisation of the Composites

**Figure 3:**
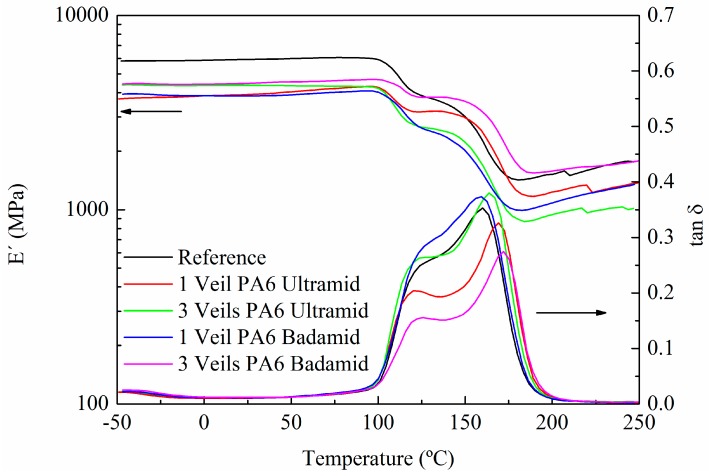
Storage modulus and tan δ of composites cured at a temperature of 90 °C.

The DMTA test was employed to investigate the elastic stiffness and damping (energy dissipation) term of the composites. The storage modulus (E’) and tan δ evolution with the temperature of the composites reinforced with one and three nanofibre veils of PA6 Ultramid and PA6 Badamid are shown in Figure 3. In the zone below the curing temperature, corresponding to the vitreous state of the composite, it seems that the veil entailed a loss in the value of E’, but it should be considered that the resin was not fully cured at this temperature. Reaching 100 °C, a decrease in the storage modulus was observed, which corresponded to the glass transition interval of the matrix that was partially cured at 90 °C. When the temperature increased, the curing process was restarted, slightly increasing the storage module. As the temperature continued to rise, the curing process finished, and the material reached its rubbery phase. Regarding the tan δ, two peaks were observed, the first above 100 °C was related to the curing process to which the samples had been subjected. As the temperature increased, the system continued cross-linking, and a second peak was obtained above 150 °C, which corresponded to the *T*g_∞_ of the system. The peaks that corresponded to the modified composites with veils seemed to move slightly towards higher temperatures, indicating a higher degree of cross-linking.

The flexural strength and deformation of the reference samples and the composites reinforced with electrospun nanofibre veils were measured, and the results are shown in Table 3. As expected, no delamination was observed in the composites.

From these results, it is clear that both PA6 Ultramid and PA6 Badamid nanofibrous veils toughened the composite laminates considerably, leading to an increase in their flexural strength of 19.7% for the composites that were modified with one veil of PA6 from Ultramid, and 42.4% for the composites that were modified with one veil of PA6 from Badamid. This increase was slightly lower for the composites with three veils for both types of PA6, indicating that the veils on the exterior faces did not contribute positively to the improvement of the flexural mechanical properties. It could even be said that the efficiency of the intermediate veil of the composite was reduced to a certain extent. Comparing the differences observed due to the different polyamides used, it can be seen that the percentage of improvement that was obtained with the use of the PA6 Badamid veils was higher than that observed in the composites manufactured with the PA6 Ultramid veils. The main reason is the different nature of both materials. The mechanical properties of the PA6 Ultramid are lower than those of Badamid PA6, as Badamid PA6 was designed for the production of engineering pieces by injection. In contrast, PA6 Ultramid was designed for the manufacture of textile filaments. Furthermore, as it has previously observed in the characterisation of the nanofibrous veils, the crystallinity of the PA6 Badamid veils was greater than that of the PA6 Ultramid veils, which could contribute to the observed improvement of their properties.

**Figure 4:**
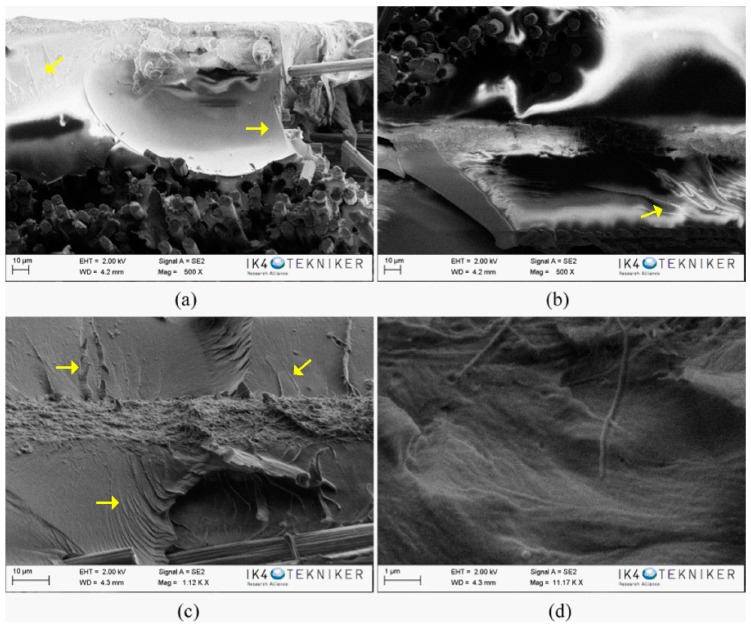
SEM images of: (**a**) reference composite; (**b**,**c**) composite with PA6 Badamid and PA6 Ultramid veil, respectively; and (**d**) a magnification of the veil area in PA6 Ultramide composite. The arrows show the propagating cracks on the composite.

After flexural tests, the fracture surfaces of the specimens were characterised by SEM. The SEM image of the reference composite specimen showed, in the resin-rich region (Figure 4a), the typical brittle fracture surface of thermostable matrices, explaining its poor tenacity. On the contrary, in the composites with an interlaminar veil of PA6 Badamid (Figure 4b) and PA6 Ultramid (Figure 4c), the fracture waves of the resin ended in the veil. It seems that the veils prevented the propagation of the crack through the polymeric matrix. This difficulty of propagation was responsible for the higher values of flexural strength in the composites reinforced with veils, contributing to the improvement of their mechanical properties. For the composite with the PA6 Badamid veil, (Figure 4b), the veil seemed to be more integrated in the epoxy matrix. In the composite with PA6 Ultramid, at higher magnifications (Figure 4d), nanofibres with diameters around 120 nm could be even observed. These values were slightly higher than those found for the diameters of the nanofibres in the veil, which could indicate the impregnation of the fibres with the resin.

**Figure 5:**
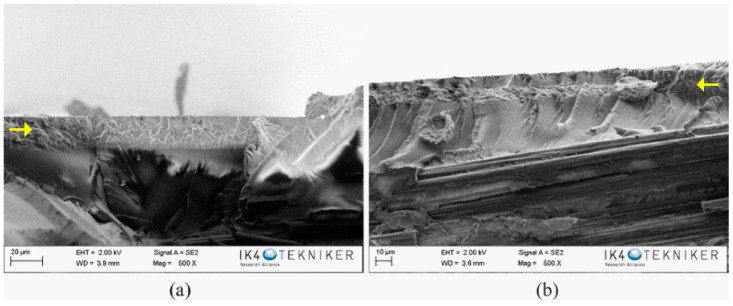
SEM images of composites with the outer veils (**a**) PA6 Badamid and (**b**) PA6 Ultramid. The arrows show the presence of the outer veils on the composites.

However, while the interlaminar veil clearly stopped the crack propagation, the outer veils (Figure 5) did not seem to contribute to avoid it, since many cracks continued their advance through the matrix. This would explain why the presence of external veils did not improve the mechanical properties of the composites that were subjected to flexural analysis. In addition, a difference in the position of the outer veils is appreciated as being in some cases more external (Figure 5a) and in other cases having more resin on the external part (Figure 5b). The infusion process can result in composite materials with a very thin external face of resin or an outer face formed by the resin-impregnated veil. Moreover, it is possible that both configurations coexist in the same composite. This could explain the small deviations in the mechanical and dynamic–mechanical properties that were observed when comparing the different systems.

**Figure 6:**
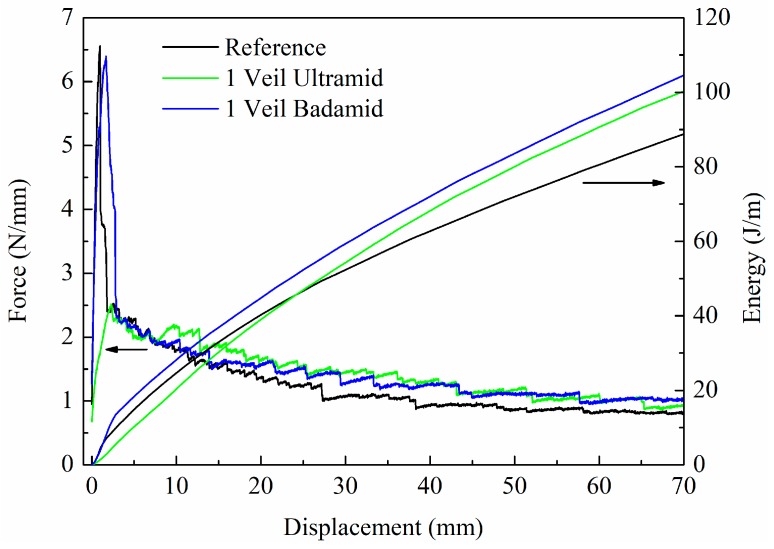
Mode-I test results. Load and mechanical energy normalised to specimen width as a function of displacement.

The fracture behaviour of the composites was carried out through mode-I and mode-II tests on specimens with 14 layers of carbon fibre fabric and an interlaminar veil. The experimental results of the G_IC_ tests, load–displacement and mechanical energy–displacement graphs, that were obtained for the specimens with the two types of PA6, Ultramid and Badamid veils, as well as the results of the reference specimen, are presented in Figure 6, and the results are reported in Table 4. Both the force and the mechanical energy values were normalised to specimen width. The graph shows the typical drop in load for the reference and PA6 Badadmid composites, which was associated to an artificially induced pre-crack that was partially glued. After overtaking this initial displacement, the three composites had a similar behaviour until displacements of 8 nm and 15 mm, where the load values of the PA6 Ultramid and Badamid composites had a greater magnitude with respect to the reference composite, respectively. Such a trend is confirmed by the propagation mechanical energy calculated by means of the integration the load displacement diagram.

In the mechanical energy diagram (Figure 6), it is interesting to note that at low displacement values, the three composites have relatively similar energy trends. By increasing the displacement for the composite with PA6 Badamid, the energy observed is always greater than that of the reference and the composite with PA6 Ultramid veil. For the composite with PA6 Ultramid, this energy is slightly lower until displacements of 20 mm. Thereafter, the composite is able to support a higher force value with respect to the reference composite. Such behaviour can be related to the presence of the veil layer that tends to obstacle the crack propagation in mode I. On the contrary, the veil presence at the crack-opening interface improves the mechanical energy that is absorbed during the crack propagation test. It is estimated that the presence of the veil in the crack-opening interface contributes to an increment in the absorbed energy of about 8.6% for the composite with the PA6 Ultramid veil and approximately 14.8% for the PA6 Badamid composite with respect to the reference composite calculated at a value displacement of 40 mm. Finally, it is observed that the presence of the interleaved veil has a measurable influence also on the critical strain energy release rate, with the G_IC_ value in the case of the composites with the PA6 Ultramid and PA6 Badamid veils being about 20% and 44% higher, respectively, with respect to the reference composite.

**Figure 7:**
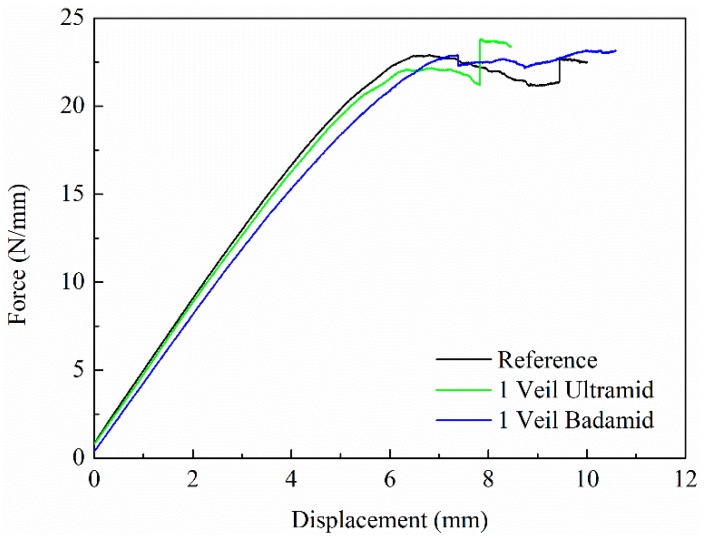
Mode-II test results. Load normalised to specimen width as a function of displacement.

The results of the mode-II experiments that were obtained for the specimens with the two types of PA6 veils, as well as the results of the reference specimens, are shown in Figure 7. The force values were normalised to specimen width. It should be noted that the veils do not influence the stability of the composites; thus, the initial linear part of the curves of the reference composite and the composites with PA6 veils practically overlap. However, the effect of the veil on the load capacity is visible as soon as the crack begins to propagate.

Table 5 collects the values of the mode-II interlaminar fracture toughness (GIIC) of the composites. Comparing the G_IIC_ value obtained for the reference composite and the PA6 Ultramid composite, no significant improvement is observed. However, for the PA6 Badamid composite, this value increases by 16.8% compared to the reference composite.

**Figure 8:**
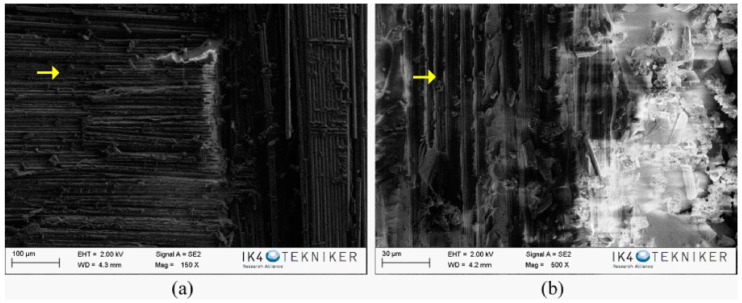
Specimen after fracture test of reference composite. The arrows show the presence of the carbon fibres on the composites.

**Figure 9:**
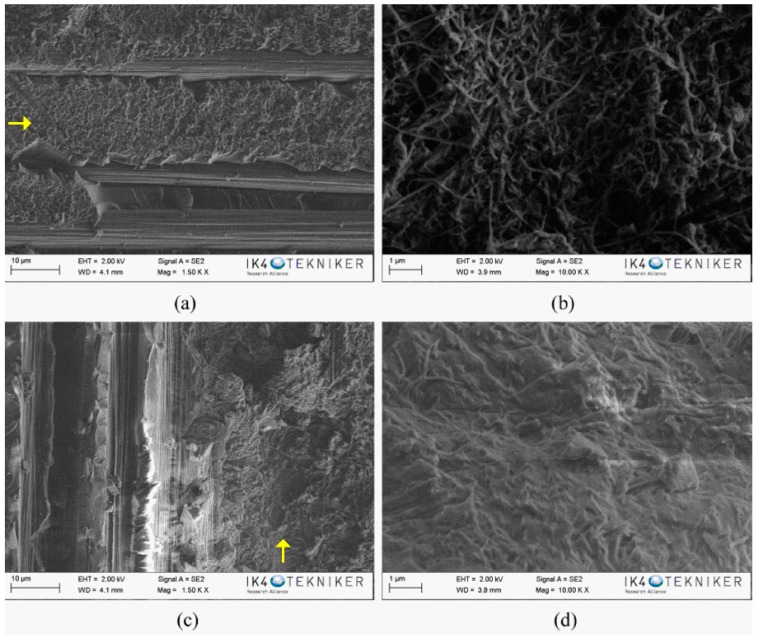

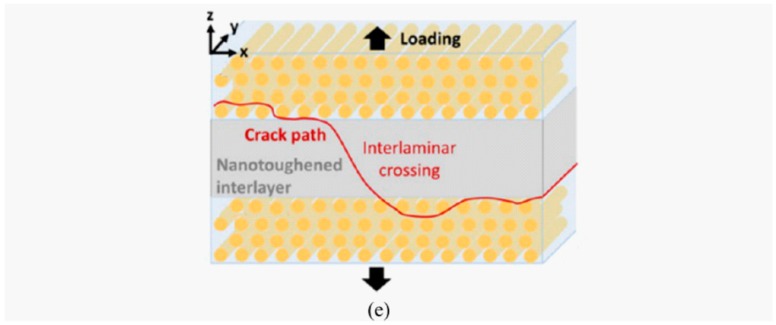
SEM images of the fracture surface of the composite specimens with PA6 veils (**a**,**b**) Ultramid and (**c**,**d**) Badamid. (**e**) Schematic view of the delamination path in reinforced composites under mode I testing. Reprinted (adapted) with permission from Daelemans, L.; van der Heijden, S.; De Baere, I.; Rahier, H.; Van Paepegem, W.; De Clerck, K [43]. Copyright (2018) American Chemical Society. The arrows show the presence of the veils on the carbon fibres.

The fracture surfaces of the composite specimens after the mode-II fracture test have also been characterised by SEM. In the SEM image of the reference, as seen in Figure 8, carbon fibres and resin-rich zones are observed. In the SEM images corresponding to the fracture of the composites with a veil of PA6 Ultramid and PA6 Badamid, as shown in Figure 9a,c, the veil deposited on the carbon fibres can be observed in some areas. This indicates that the surface of the veils remains in both sides of the composite specimen, and that the crack propagates partially between the carbon fibre and the veil, as shown schematically in Figure 9e. At higher magnifications (Figure 9b,d), the nanofibres that form the veils of both PA6 Ultramid and PA6 Badamid can be seen. It was observed, as was verified in the SEM images made after the bending test, that the PA6 Badamid veil seems to be more integrated in the resin than the PA6 Ultramid veil, as its nanofibres are distinguished more clearly. This could be ascribed to the small differences in the porosity and porous distribution and therefore to the resin impregnation. This could contribute to the improvement in mechanical properties, which are the same as the higher crystallinity of the PA6 Badamid veil compared to PA6 Ultramid.

## 4. Conclusions

This paper investigated the effect of the incorporation of two different type of electrospun polyamide 6 nanofibre veils on the mechanical behavior of carbon fibre/epoxy composites manufactured by a vacuum infusion process. The veils obtained from both types of polyamides, Badamid^®^ B70 and Ultramid^®^ B24 N 03, which present similar fibre diameter and aerial density but different crystallinity (38.2% for PA6 Badamid and 26.6% for PA6 Ultramid), were incorporated in different composite-laminated positions.

The results indicated that the incorporation of polyamide nanofibre veils increases their mechanical properties. For composites with one PA6 nanofibre veil between the carbon fibre plies, the stress at failure during the flexural mechanical tests increased by 19.7% and 42.4% for composites modified with PA6 Ultramid and PA6 Badamid, respectively. The analysis of the fractured surfaces, carried out by SEM, indicated that the veil hindered the crack propagation in the composites. For composites with three veils, one in the middle and the other two on the external layers of the composites, the stress at the failure improvement was slightly lower than for the composites with one interleaved veil, indicating that the external veils did not contribute to the observed improvement. This statement has been checked by analysing the crack propagation by SEM. In both cases, the veils from Badamid, with higher crystallinity, showed better results than the veils from Ultramid. Furthermore, the fracture toughness analysis showed that G_IC_ value increased by 20% and 44% for composites modified with a veil of PA6 Ultramid and PA6 Badamid, respectively, whereas the G_IIC_ values only increased slightly for the composite modified with the PA6 Badamid veil. This increment is due to the crack propagation across the PA6 veil, which resulted in the high-energy absorption of the veil.

Summarising, the inclusion of electrospun polyamide 6 nanofibre veils on the carbon fibre/epoxy composites resulted in a significant improvement in mechanical properties, in relation to both flexural and fracture toughness, without an increase in laminate thickness or weight, and maintaining or slightly increasing the glass transition temperature of the composite.

## Figures and Tables

**Table 1:** Diameter and areal weight density of PA6 nanofibres.

Sample	Diameter (nm)	Weight (g·m^−2^)
PA6 ULTRAMID NFs	60–100	1.94
PA6 BADAMID NFs	60–130	2.23

**Table 2:** Melting temperatures and percentages of crystallinity.

Material	First Scan			Second Scan		
	*T*_m_ (°C)	*ΔH*_m_(J/g)	*X*_c_ (%)	*T*_m_ (°C)	*ΔH*_m_(J/g)	*X*_c_ (%)
ULTRAMID Pellet	225.1	107.1	46.5	221.0	59.0	25.6
ULTRAMID Veil	224.3	61.2	26.6	220.1	56.1	24.4
BADAMID Pellet	224.1	95.4	41.5	220.3	76.6	33.3
BADAMID Veil	225.0	87.8	38.2	219.9	81.4	35.4

**Table 3:** Results obtained after flexural tests on composites with different PA6 veils.

Sample	σ_max_ (MPa)	Δσ_max_ (%)	δ_max_ (%)
Reference	375.5 ± 33.2		2.2 ± 0.2
1 veil PA6 Ultramid	449.5 ± 10.8	19.7	2.1 ± 0.0
3 veil PA6 Ultramid	415.4 ± 23.8	10.6	2.1 ± 0.2
1 veil PA6 Badamid	534.6 ± 28.9	42.4	2.3 ± 0.0
3 veil PA6 Badamid	502.3 ± 48.5	33.8	2.1 ± 0.1

**Table 4:** Mode I results.

Sample	Energy (J/m)	Δ%	G_IC_ (J/m^2^)	Δ%
Reference	62.7		389 ± 12.8	
PA6 Ultramid	68.1	8.6	466 ± 73.0	20.0
PA6 Badamid	72.0	14.8	560 ± 72.3	44.0

**Table 5:** Mode II results.

Sample	G_IIC_ (J/m^2^)	Δ%
Reference	2536.8 ± 257.7	
PA6 Ultramid	2544.0 ± 304.0	0.3
PA6 Badamid	2970.9 ± 526.0	16.8
